# Transcriptomic Analysis of Conserved Telomere Maintenance Component 1 (CTC1) and Its Association with Leukemia

**DOI:** 10.3390/jcm11195780

**Published:** 2022-09-29

**Authors:** Saadiya Zia, Netasha Khan, Komal Tehreem, Nazia Rehman, Rokayya Sami, Roua S. Baty, Faris J. Tayeb, Majed N. Almashjary, Nouf H. Alsubhi, Ghadeer I. Alrefaei, Ramla Shahid

**Affiliations:** 1 Department of Biosciences, COMSATS University Islamabad (CUI), Islamabad 45550, Pakistan; 2 Department of Biochemistry, University of Agriculture Faisalabad, Faisalabad 38000, Pakistan; 3 Department of Food Science and Nutrition, College of Sciences, Taif University, P.O. Box 11099, Taif 21944, Saudi Arabia; 4 Department of Biotechnology, College of Science, Taif University, P.O. Box 11099, Taif 21944, Saudi Arabia; 5 Department of Medical Laboratory Technology, Faculty of Applied Medical Sciences, University of Tabuk, Tabuk 47713, Saudi Arabia; 6 Department of Medical Laboratory Sciences, Faculty of Applied Medical Sciences, King Abdulaziz University, Jeddah 22254, Saudi Arabia; 7 Hematology Research Unit, King Fahd Medical Research Center, King Abdulaziz University, Jeddah 22254, Saudi Arabia; 8 Biological Sciences Department, College of Science and Arts, King Abdulaziz University, Rabigh 21911, Saudi Arabia; 9 Department of Biology, College of Science, University of Jeddah, P.O. Box 80327, Jeddah 21589, Saudi Arabia

**Keywords:** telomere modulating genes, telomere length, acute lymphoblastic leukemia, risk biomarker

## Abstract

Telomere length (TEL) regulation is important for genome stability and is governed by the coordinated role of shelterin proteins, telomerase (TERT), and CST (CTC1/OBFC1/TEN1) complex. Previous studies have shown the association of telomerase expression with the risk of acute lymphoblastic leukemia (ALL). However, no data are available for CST association with the ALL. The current pilot study was designed to evaluate the CST expression levels in ALL. In total, 350 subjects were recruited, including 250 ALL cases and 100 controls. The subjects were stratified by age and categorized into pediatrics (1–18 years) and adults (19–54 years). TEL and expression patterns of CTC1, OBFC1, and TERT genes were determined by qPCR. The univariable logistic regression analysis was performed to determine the association of gene expression with ALL, and the results were adjusted for age and sex in multivariable analyses. Pediatric and adult cases did not reflect any change in telomere lengths relative to controls. However, expression of CTC1, OBFC1, and TERT genes were induced among ALL cases. Multivariable logistic regression analyses showed association of CTC1 with ALL in pediatric [β estimate (standard error (SE)= −0.013 (0.007), *p* = 0.049, and adults [0.053 (0.023), *p* = 0.025]. The association of CTC1 remained significant when taken together with OBFC1 and TERT in a multivariable model. Furthermore, CTC1 showed significant association with B-cell ALL [−0.057(0.017), *p* = 0.002) and T-cell ALL [−0.050 (0.018), *p* = 0.008] in pediatric group while no such association was noted in adults. Together, our findings demonstrated that telomere modulating genes, particularly CTC1, are strongly associated with ALL. Therefore, CTC1 can potentially be used as a risk biomarker for the identification of ALL in both pediatrics and adults.

## 1. Introduction

Telomeres, the DNA-protein complexes present at the end of chromosomes, are responsible for maintaining chromosomal integrity by preventing chromosomal end-end fusion and dicentric chromosome formation [1]. Telomeric repeats are lost after every cell division, leaving behind 3′ single-stranded overhangs (100–400 nucleotides) at the chromosomal ends. When telomeres become critically short, they lose the protective protein capping and undergo senescence and apoptosis [2]. Telomere-associated proteins include the shelterin complex, which consists of six proteins, TRF1, TRF2, RAP1, TPP1, POT1, and TIN2, that bind with TTAGGG tandem hexanucleotide telomeric repeats [3]. These proteins shield the chromosomal ends from exonuclease action and prevent end-to-end chromosomal fusion [4]. 3′single-stranded overhangs of the telomere fold back to interact with the proximal double-stranded telomeric region, forming a structure called T-loop. This conformation protects the telomere from telomerase action and impedes activation of DNA damage response, thereby preventing cellular senescence [5]. A telomeric sequence is not only species-specific, but every organism has its own characteristic telomeric sequence. In humans, the telomere length usually ranges from 10–15 kb, and this variability depends on gender, cells, age, and origin [6,7]. Somatic cells undergo more telomeric loss (50–200 bases lost/cell division) as compared to telomere synthesis [8].

Telomerase, a ribonucleoprotein absent in somatic cells but highly expressed in germ cells, stem cells, embryonic cells, immortalized cell lines, and tumor cells, is responsible for the continued and unlimited proliferative potential of cells [9]. Telomerase enzyme has two essential components; the RNA component (TERC/hTR) provides a template for the addition of nucleotides to the 3′ telomeric overhangs. The other component, telomerase reverse transcriptase (TERT), catalyzes the addition of TTAGGG hexanucleotide repeats for telomere elongation [10]. Reactivation of telomerase occurs in 90% of tumors for telomere length regulation and equips cancer cells with unlimited proliferation activity [11]. Telomerase thus prevents telomere shortening by stabilizing shorter telomeres [12]. In cancer cells, telomerase activation is often associated with promoter mutations and sporadic mutations near the transcription start site that creates a binding site for the ETS transcription factor [13].

**Figure 1:**
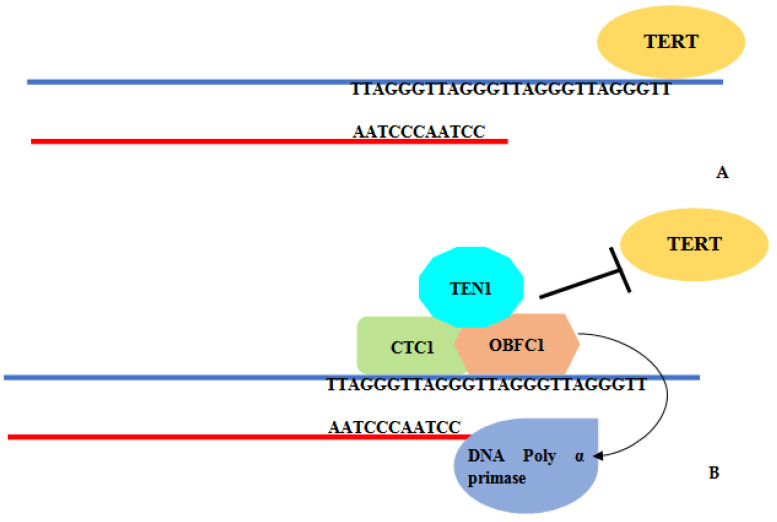
(**A**) “G” strand extension by telomerase. (**B**) CST complex (CTC1, OBFC1, TEN1) recruits DNA Pol α primase for elongation of C strand and inhibits telomerase binding.

Apart from the shelterin complex, telomeres gain additional protection from the CST complex, which consists of conserved telomere capping protein1 (CTC1/AAF-132), Oligonucleotide/oligosaccharide-binding fold containing protein1 (OBFC1/STN1) and Telomeric pathways in association with STN1 (TEN1). Human CTC1 is analogous to yeast cdc13 [14]. The human CST complex has been characterized as a telomere replication factor. In mammals, 20% of the CST foci have telomeric-specific roles, while 80% are localized in the nucleus with non-telomeric functions [15]. The components of the CST complex are involved in “C strand fill in” by recruitment of DNA pol α primase [2] (Figure 1). CST binds with telomeric DNA in a sequence-independent manner [16]. The components of the CST complex assemble at and sequester the 3′ telomeric overhangs and prevent the binding of telomerase enzyme [17]. The structural similarity of the CST components (OBFC1 and TEN1) with RPA (replication protein A) suggests the role of the CST complex in replicative stress [17]. During DNA damage, uncoupling of polymerase and helicase is followed by binding of the CST complex with stalled replication fork. CST recruits DNA polymerase α primase to reinitiate replication [18,19]. Any impairment in CST components leads to replication stalling, dysregulation of telomere replication, and genome instability due to irreparable DNA damage [20]. The conservation of the CST complex in yeasts, plants, and mammals highlights the significance of this complex in telomere protection and modulation.

Several studies have shown an association of telomere modulating or regulating genes with different diseases and cancer. Luo et al. [21] described CTC1 as a possible target and predictive biomarker in melanoma treatment. A potential association has been observed between the genetic variants of OBFC1 and cancers such as laryngeal, prostate, glioma, and chronic lymphocytic leukemia [22]. Similarly, mutated TERT is used as a diagnostic biomarker in bladder cancer and a prognostic biomarker in breast and colorectal cancer [23,24]. In a recent study, Wang et al. [25] identified the CST complex as a predictive biomarker for immune checkpoint blockade in cancer. 

The aim of the current pilot study was to find the association of telomere-modulating genes (CTC1, OBFC1, TERT) with ALL and to see if they could be used as a risk biomarker for early identification of ALL.

## 2. Materials and Methods

### 2.1. Study Participants

The research was conducted in accordance with the Helsinki Declaration and was approved by the Ethics committee of COMSATS University Islamabad (5 March 2015) and participating hospitals Institute of Nuclear Medicine and Oncology Lahore (INMOL) and Pakistan Institute of Medical Sciences (PIMS), Islamabad. All participants provided written informed consent.

Initially, in the screening process, participants newly diagnosed with ALL, based on the bone marrow biopsy and immunophenotyping results, were referred by the oncologists for the study. Later, only those participants were selected for sample collection that conformed to the selected inclusion and exclusion criteria. Inclusion criteria included newly diagnosed ALL cases. Subjects previously treated for any carcinoma, child-bearing women, first-degree relatives, and those having any other disease were excluded from the study. The demographic data such as age, gender, and blood groups were collected from the patient hospital record. Healthy individuals without any disease, which served as a control, were also recruited for the study.

Blood samples of 250 ALL cases and 100 healthy individuals with age ranging from 1–54 years were collected. The study participants were divided into two groups on the basis of age as pediatric (1–18 years) and adult (19–54 years) groups. 

### 2.2. DNA and RNA Extraction

The whole blood of cases and controls were subjected to RBCs lysis to obtain white translucent WBCs pellet [26]. Once the WBCs pellet was obtained, samples were processed for DNA and RNA isolation. RNA extraction was carried out by trizol method according to the manufacturer’s protocol (Invitrogen, Waltham, MA, USA). Genomic DNA was isolated by the modification of trizol and phenol chloroform method. Quantification of both DNA and RNA was performed by nanodrop (IMPLEN, München, Germany), and yield was checked by gel electrophoresis. 

### 2.3. Telomere Length Measurement

For telomere length measurement, real-time PCR (48-well ABI Step One) was performed according to Cawthon’s method [27]. A total of 35 ng of the isolated DNA was used for a 20 μL reaction. The sequence of primers for telomere length (TEL) and β-globin (single copy gene) was represented in Table 1. Telomere length was calculated in terms of T/S ratio, which represented telomere length relative to single copy gene, i.e., β-globin. 

### 2.4. Expression Analysis of CTC1, OBFC1, and TERT by qPCR

500 ng cDNA was prepared from the isolated RNA by MMLV-RT (Thermo Fisher, Waltham, MA, USA). Gene expression analysis of CTC1, OBFC1, TERT, and β-globin wascarried out by using qPCR master mix (Thermo Fisher, USA) on ABI StepOne detection system (Thermo Fisher Scientific, USA). β-globin was used as an endogenous control. The sequence of primers for CTC1, OBFC1, and TERT genes is represented in Table 1. The thermal cycling conditions were 95 °C initial denaturation for 5 mins followed by 40 cycles of 95 °C for 45 secs, CTC1 (52 °C), OBFC1 (48 °C), TERT (58 °C), β-globin (58 °C) for 30 secs, 72 °C for 30 secs. Data acquisition was performed at the extension step. The expression of CTC1, OBFC1, and TERT relative to the endogenous control β-globin gene was analyzed by the 2^−ΔΔCt^ method.

### 2.5. Statistical Analysis 

The statistical analyses were performed in R v4.0.3 [28]. The categorical variables are represented as N (%), and χ^2^ was applied to determine the differences between cases and controls. The continuous variables are represented as Mean (SE). As ΔCt values of CTC1, OBFC1, and TERT genes were not normally distributed (Figure S1A–F); therefore, Mann–Whitney U test was applied to compare cases with controls. Pearson correlation coefficients were also calculated between the genes CTC1 and OBFC1, CTC1 and TERT, OBFC1 and TERT, and between all these three genes and TEL.

Multiple models were applied to determine the association of gene expression with ALL by using ΔCt values. In both pediatric and adult groups, univariable logistic regression analyses were performed to determine association of CTC1, OBFC1, and TERT with ALL (Model 1), and results were adjusted for age and sex (Model 2). Combined effect of all the genes with ALL was assessed (Model 3), and these results were also adjusted for age and sex (Model 4). 

Univariable logistic regression analysis was applied to determine the association of TEL with ALL (Model 5). In order to determine the effect of these three genes and TEL with ALL, multivariable analyses were performed (Model 6: ~TEL+ CTC1+ OBFC1+ TERT). These results were then adjusted for age and sex (Model 7). The association of telomere modulating genes with ALL immunophenotypes was also studied by univariable logistic regression analysis, and results were then adjusted for age and sex. The combined association of all the genes with ALL subtypes was also determined. The *p* values <0.05 were considered statistically significant.

## 3. Results

### 3.1. Cohort Description

The demographic characteristics of the cases and controls are summarized in Table 2. In total, 350 subjects were recruited, including 250 cases and 100 controls. The cases were subdivided into pediatric group (n = 185) and adult group (n = 65). Ahigher prevalence of ALL was observed in pediatric ALL cases (74%) than in adults (26%) (*p* < 2.20 × 10^−16^), respectively. In pediatrics, more males had the disease and were significantly older (*p* = 0.001) as compared to controls. While in adults, there was no significant difference in age between cases and controls.

The results also showed that the B blood group was more common among the cases as compared to other blood groups, as 47.2% of pediatric and 34.0% of adult cases had the B blood group. The majority of the cases had B-cell ALL. The prevalence of B-cell ALL was 77% in pediatric and 72.3% in adult cases. 

The cytogenetic data of ALL cases showed chromosomal translocations, deletions, duplications, and additions (Table 2). Hyperdiploidy with other abnormalities was observed in both pediatric and adult B-cell ALL, while pediatric T-cell ALL showed mosaicism and hypodiploidy.

### 3.2. Telomere Length Maintenance in ALL Cases

**Figure 2:**
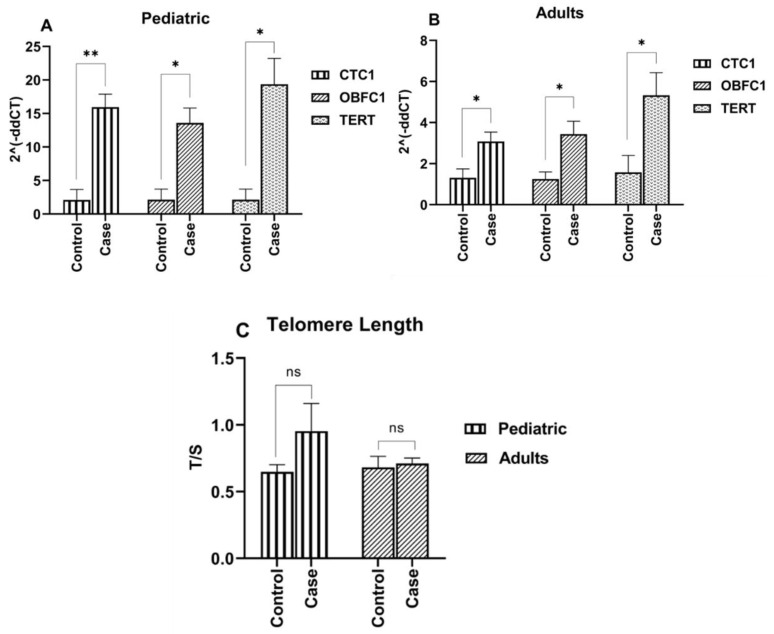
(**A**) Telomere length in pediatric and adult control and ALL groups expressed as T/S ratio. (**B**,**C**) Expression of telomere modulating genes CTC1, OBFC1 and TERT in (**B**) pediatric (1–18 years) and (**C**) adult (19–54 years) groups. Error bars represent standard error of mean. n.s = non-significant *p* value > 0.05, * = *p* value < 0.05, ** = *p* value ≤ 0.01.

In the present study, we measured TEL in ALL cases and controls and represented it in the form of the T/S ratio (Figure 2A). No significant difference in the TEL between the cases and control was noted in the pediatric (*p* = 0.33) and adult groups (*p* = 0.4). It seems that TEL in ALL cases was rather maintained at a lower normal range. 

### 3.3. Elevated Expression of Telomere Modulating Genes (CTC1, OBFC1 and TERT) in ALL Cases

The expression of telomere modulating genes CTC1, OBFC1, and TERT measured by qPCR in pediatric and adult cases and control samples was represented in Figure 2B,C. Expression of all the genes CTC1, OBFC1, and TERT wasinduced in pediatric and adult cases relative to the controls. The expression pattern of TERT was coherent withthat of CTC1 and OBFC1 genes. CTC1 expression was induced 14.6 (3.00 × 10^−4^) and 2.8 (*p* = 0.040) folds in pediatric and adult leukemia cases. Similarly, OBFC1 showed 12.0 (*p* = 0.024) and 3.0 folds (*p* = 0.040) and TERT 16.7 (*p* = 0.016) and 4.6 (*p* = 0.039) folds elevation in the expression in pediatric and adult cases relative to the controls. Thus, significant upregulation of telomere modulating genes was observed in all ALL cases.

### 3.4. Telomere Modulating Genes Expression and ALL Immunophenotypes

**Figure 3:**
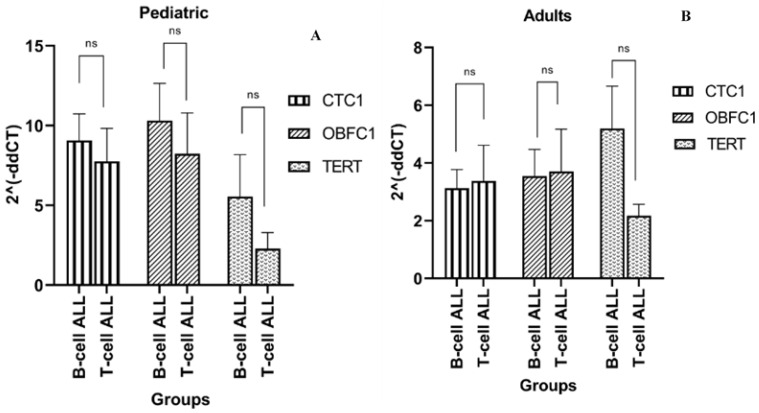
Expression of telomere modulating genes in (**A**) pediatric (1–18 years) and (**B**) adult (19–54 years) ALL cases having B-cell and T-cell ALL. Error bars indicate standard error of mean. n.s represents non-significant *p* value > 0.05. In both pediatric and adult groups, significant difference between genes expression and ALL immunophenotypes was not noted.

The expression pattern of telomere modulating genes CTC1, OBFC1, and TERT was also measured in ALL cases having different immunophenotypes (Figure 3A,B). It was observed that in both pediatric and adult groups, although the expression of the genes was elevated in B-cell ALL as compared to T-cell ALL, the difference was not statistically significant. 

### 3.5. Correlation among the Telomere Modulating Genes 

**Figure 4:**
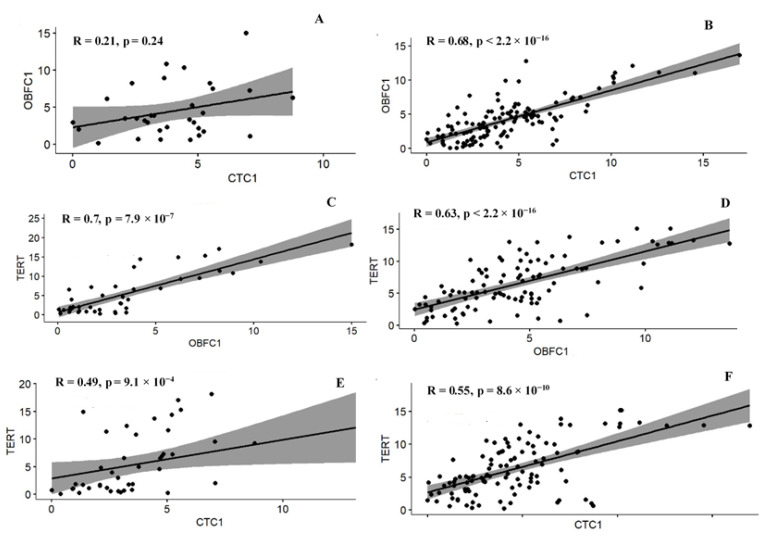
Correlation between genes (**A**,**B**) CTC1 and OBFC1, (**C**,**D**) OBFC1 and TERT, (**E**,**F**) CTC1 and TERT. (**A**,**C**,**E**) = adult (19–54 years) groups and (**B**,**D**,**F**) = pediatric (1–18 years) groups. Positive correlation has been noted between the genes in both pediatric and adult groups.

Strong correlation was observed between OBFC1 and TERT genes in the adult group (r = 0.70; *p* = 7.9 ×10^−7^) (Figure 4A–F). However, the same genes OBFC1 and TERT (r = 0.63; *p* < 2.2 ×10^−16^) showed moderate while strong correlation was noted between CTC1 and OBFC1 (r = 0.68, *p* < 2.20 × 10^−16^) genes in pediatrics. In adult group, the correlation between CTC1 and OBFC1 was nonsignificant (r = 0.21; *p* = 0.24). We also observed moderate correlation between CTC1 and TERT in both adult (r = 0.49; *p* = 9.1 × 10^−4^) and pediatric (r = 0.55; *p* = 8.6 × 10^−10^) groups. However, a negative and nonsignificant correlation has been noted between the selected genes and telomere length (Figure S2A–F).

### 3.6. Association of Genes Expression with ALL

The univariable logistic regression analysis showed association of CTC1 with ALL in both pediatric [β-estimate (SE) = −0.045 (0.009), *p* = 3.40 × 10^−6^] and adult [0.071 (0.022), *p* = 0.002] groups. These associations remained significant after adjusting results with age and sex in multivariable logistic regression analysis (Table 3A,B)).

In the pediatric group, the multivariable analysis with all three genes in the model, a significant association was observed only in the case of CTC1: [−0.042 (0.016), *p* = 0.010)]. This association also remained significant after adjusting for age and sex (Table 3C). Similarly, in adults, in the multivariable analysis for determining the combined effect of genes on ALL, the association of CTC1 remained significant even after adjusting for age and sex [=0.105 (0.037), *p* = 0.009) (Table 3D). 

### 3.7. Association of TEL with Leukemia

Univariable logistic regression analysis revealed no association of TEL with ALL in pediatrics. The results remained unchanged after adding all genes in Model 6 and adjusting for age and sex in Model 7 (Table 4A–C). These results are consistent with the correlation coefficients calculated between the genes and TEL (Figure S2A–F). Similarly, in adults, a significant association of TEL with ALL was not observed (Table 4D–F).

### 3.8. Association of Genes Expression with ALL Immunophenotypes 

Univariable logistic regression analysis in pediatric group showed significant association of CTC1 gene with B-cell ALL [β-estimate (SE) = −0.057 (0.017), *p* = 0.002)] and T-cell ALL [−0.050 (0.018), *p* = 0.008)]. However, these associations became nonsignificant when adjusted for age and sex in both cases. In adults, the significant association of the TERT gene [−0.093 (0.043), *p* = 0.036)] with B-cell ALL was observed, and it remained significant after adjusting for age and sex. When the combined association of genes with B-cell ALL was observed in pediatric group, CTC1 [−0.057 (0.023), *p* = 0.016)] and TERT [β-estimate (SE) = 0.047 (0.023), *p* = 0.040)] showed significant associations. However, only the CTC1 association remained significant after adjusting forage and sex.

Univariable logistic regression showed no association of the combined effect of all three genes with T-cell ALL in pediatrics. However, these results became significant in multivariable logistic regression analysis adjusted with age and sex. In contrast, no association was observed when the combined effect of all the genes with B and T-cell ALL was studied in adults (Table 5).

## 4. Discussion

The current pilot study was designed to evaluate the association of the CST complex genes with ALL. Our results demonstrated upregulation of telomere modulating genes, including CST complex genes (CTC1 and OBFC1) and TERT expression in ALL cases in spite of no change in the telomere lengths relative to controls. We observed a significant association of CTC1 with ALL in both pediatric and adult cases. 

In this study, we noted that almost 74% of the cases belong to the pediatric group. Increased incidence of ALL in the pediatric group could be related to the genetic changes and development of preleukemic clones in fetal life [29]. We also observed that males were predominately more common in both the pediatric (67%) and adult (78%) groups than the females. The same data of increased ALL incidence in males have been reported internationally [30]. Our results demonstrated that blood group “B” was more common in pediatric (47.2%) and adult (34%) cases as compared to other blood groups. However, some studies have shown the occurrence of O and AB blood groups in ALL cases [31,32]. Among ALL subtypes, we have noted that B-cell ALL was more prevalent than T-cell ALL. Several studies have shown that T-cell ALL is less prevalent and represents 20–25% of ALL cases [33,34]. The cytogenetic data of ALL cases showed chromosomal translocations t(9;15)(p13;q11.2), t(1;19)(q25;p13.3), t(9;22)(q34;q11.2), and hyperdiploidy with other abnormalities in B-cell ALL while hypodiploidy was noted in T-cell ALL cases. Several studies have identified the same chromosomal translocations and the presence of hypo and hyperdiploid chromosomes in ALL cases [35,36,37,38,39]. 

In the present study, telomere length was measured in both pediatric and adult ALL cases. In ALL, telomeric length is usually shorter than AML [10]. Generally, the cancer cells undergo a gradual decrease in telomere length due to increased cell proliferation and altered telomere regulatory mechanisms [24,40]. Telomere length is greatly influenced by lifestyle and disease status [41]. In certain cases, longer telomeres increase the risk of childhood cancers such as neuroblastoma and acute lymphoblastic leukemia [42]. Our results demonstrated that there was no significant difference in the telomere length in cases as compared to controls in both pediatric and adult groups. Contrastingly, in several studies, telomere shortening has been reported in acute leukemia [10,43,44]. However, few studies have also reported telomere length maintenance in acute myeloid leukemia, as leukemia cells bypass telomere crisis due to the upregulation of telomerase activity [45]. 

In order to investigate the telomere length maintenance in ALL cases, the expression of telomere modulating/regulating genes CTC1, OBFC1, and TERT was measured. CTC1 and OBFC1 are the components of the CST complex that helps the telomeres to swap between the capping and elongation state. CTC1 and OBFC1 are important for telomere replication by recruiting DNA Pol α primase for C-strand synthesis [46]. They terminate telomerase activity once the 3′ overhangs of the Gstrand are elongated [47]. We found induced CTC1, OBFC1, and TERT expression in pediatric and adult cases. A comparison of telomere modulating gene expression between pediatric and adult cases clearly indicated that the increased expression of telomere genes in the pediatric group is due to increased telomere attrition. Telomeres undergo extensive shortening during the first few years of life and then gradually shorten with age progression [48]. High TERT expression has been detected in normal blood cells of children as compared to adults. Our data showed more telomerase expression in the pediatric group than in the adults. This is consistent with the findings that the expression of telomerase decreases with age in lymphocytes [49]. In pediatric ALL, TERT expression is usually very high at the time of diagnosis and decreases at the remission stage [42,50]. Luo et al. [21] havereported elevated CTC1 expression in radioresistant melanoma cells. Cells with CTC1 deletion experience telomere shortening [46]. Increased copy number of the CTC1 gene has been reported in osteosarcoma [51]. Similarly, the elevated expression of the CST complex genes has also been noted in ductal breast carcinoma. The OBFC1 rs9420907-C allele is significantly associated with the longer telomere length [52]. Ojha et al. [53] reported that genetic variation in OBFC1, TERT, and TERC are associated with an increased risk of chronic lymphocytic leukemia due to elongated telomere length. Elevated expression of OBFC1 has also been found in cutaneous malignant melanoma [54]. In addition to the telomeric role, the CST complex genes perform some non-telomeric roles as well. They protect double-stranded breaks from end resection and help the genome to recover from the hydroxyurea-induced replicative stress by increasing origin firing [15,55].

High TERT expression in ALL cases implies elongation of the telomeric G strand. Similarly, induced expression of CTC1 and OBFC1 was related to the telomeric C-strand synthesis and genome replication. The plausible explanation for telomere length maintenance in ALL cases relative to the controls could be the induced expression of telomere modulating genes that were preventing the telomere shortening. High telomerase activity has been detected in acute myeloid and lymphoid leukemias [45]. In 85–90% of human cancers (liver, bladder, thyroid, breast, kidney, melanoma, and glioblastoma) and cancer cell lines, telomerase is present in sufficiently high quantity to prevent telomere attrition [9,56]. Blast cells with unlimited proliferation during leukemia development usually undergo telomere attrition, but in our results, as telomere length was maintained so we speculated that elevated expression of telomere modulating genes might be responsible for telomere length maintenance in ALL cases.

The expression of CTC1, OBFC1, and TERT genes were found to be upregulated in both pediatric and adult B-cell ALL than in T-cell ALL cases but the difference was not statistically significant. Contrary to our results, Cogulu et al. [57] reported elevated TERT expression in B-cell ALL than in T-cell ALL. B-cells have longer telomeres as compared to T-cell types [7]. Some B cells show upregulated telomerase expression to support the clonal selection of cells for antibodysecretion [44].

The data of CTC1, OBFC1, and TERT gene expression in ALL cases corresponded well with their correlation results. The genes were not only upregulated in cases but were positively correlated with each other except for CTC1 and OBFC1 in the adult group. The results of the correlation coefficient showed that although the genes were correlated significantly, the correlation magnitude was not the same. CTC1 and OBFC1 showed a strong positive correlation with each other in the pediatric group. This could be because both CTC1 and OBFC1 are the subunits of the same complex that elongates the C-rich strand of the telomeres. A strong correlation was also noted between OBFC1 and TERT genes in the case of pediatric and adult groups.

As the telomere modulating genes were upregulated and correlated with each other, further, we studied their association with ALL to see if these genes could potentially be used as a risk biomarker in ALL. The association of telomere modulating genes with ALL showed an entirely different trend in both pediatric and adult cases. This might be because of different disease pattern in both groups. In the pediatric group, overexpression of genes indicated an inverse relation, while in adults, the gene expression varied proportionally with the disease risk. In the multivariable logistic models, a significant association of the CTC1 gene with ALL was noted, demonstrating that higher expression of the gene will increase the risk of ALL in adults. Statistically significant results of CTC1 association with ALL risk were also reported in pediatric cases. When we studied the combined effect of all these genes with ALL, again, only CTC1 showed a significant association with the risk of ALL in both pediatric and adult groups. The association remained significant when adjusted for age and sex. Based on the significant association of CTC1 with ALL risk, we suggested that telomere modulating gene CTC1 has thepotential to be used as a risk biomarker in ALL cases. In a similar study, it has been reported that CTC1 could be used as an independent predictive factor, as its upregulation prevented telomere shortening and increased radioresistance in melanoma cells [21]. Several studies have also highlighted the role of the CST component genes as a risk and prognostic biomarker in different cancers (lungs, breast, and gastric carcinomas) [18].

In cases (pediatric and adults), no association of telomere length was observed with ALL in the current study. Although the expression of all telomere modulating genes was induced in ALL cases, the insignificant results of multivariable logistic regression models (6B, 6E, 7C, 7F) in which association of all the genes and telomere length with ALL was studied suggested that these genes might have some non-telomeric role in ALL as well. However, in some studies, telomere attrition has been linked with the pathogenesis of ALL [43]. However, there are many other factors, both genetic and non-genetic, that influence the initiation of ALL [58,59].

To study the association of the telomere genes with different ALL immunophenotypes, we performed regression analysis. In spite of the statistically insignificant difference between B and T-cell ALL with gene expression, the association of the genes with ALL immunophenotypes was opposite in both groups. In univariable logistic regression analysis, CTC1 showed a significant association with both B and T-cell ALL. The association became insignificant when adjusted for age and sex. However, in adults’ a significant association of TERT with B-cell ALL was noted even when adjusted for age and sex. When the combined association of all the genes with B and T-cell ALL was observed, statistically significant results were observed only in pediatric groups. Among telomere modulating genes, CTC1 was the only gene that showed significant association with the ALL immunophenotypes. Although CTC1 and OBFC1 are the components of the same complex, their different association with ALL disease suggests their non-telomeric role in leukemia as well. This non-telomeric role of the CST complex genes needs to be explored in reference to ALL. As the role of the CST complex genes has not been studied before in ALL, so, we speculated that these genes might be playing some role in hematopoiesis just like shelterin proteins [60]. Among the telomere modulating genes, CTC1 was the only gene that remained significantly associated with ALL in both pediatric and adult groups. The significant association of the CTC1 gene with ALL suggested that the CTC1 gene could be used as a risk biomarker for the identification of ALL in both pediatrics and adults. However, as the age-related biology of ALL is different in pediatric and adult groups, therefore, the therapeutic approaches and outcomes vary in both groups [61]. The genetic landscape is also significantly different in pediatric and adult acute leukemia [62]. Our results also reflected that the association of CTC1 gene expression with ALL is influenced by the age and sex in both groups. As the results of expression analysis between B and T-cell ALL were not statistically significant, so we suggest that CTC1 could be a risk biomarker for ALL irrespective of the ALL immunophenotypes in both groups. 

Despite significant addition tothe present data for risk factors associated with ALL, our study also had a few limitations, such as a smaller sample size, missing data among the groups, and blood groups of controls. Therefore, we suggest replicative studies from our population for definitive conclusions. 

Conclusively, our study highlighted the association of CTC1 gene expression with ALL and its role as a potential risk biomarker. We suggested that measuring CTC1 levels in the early course of ALL may help in the immediate identification of the disease. However, replicative studies with a large sample size are required to validate this role. 

## Figures and Tables

**Table 1:** Sequence of primers.

		Primer Sequence (5′–3′)
TEL	F	GGTTTTTGAGGGTGAGGGTGAGGGTGAGGGTGAGGGT
	R	TCCCGACTATCCCTATCCCTATCCCTATCCCTATCCCT
β-globin	F	GCTTCTGACACAACTGTGTTCACTAGC
	R	CACCAACT TCATCCACGTTCACC
CTC1	F	TGAGCTGGAAAGGAAACCGT
	R	AGAAAGGCA GGACAATCGGA
OBFC1	F	TTTCACAGCTCAGCCCTAGA
	R	AAGCTCT GCACTCTGTTCTC
TERT	F	ATCAGACAGCACTTGAAGAGGGTG
	R	CCCACGACGTAGTCCATGTTCAC

TEL = Telomere length, F = forward, R = reverse.

**Table 2:** Demographic and cytogenetic characteristics of control and cases.

Cases vs. Control						
	Pediatric			Adults		
Characteristics	Controls	Cases	*p* Value	Controls	Cases	*p* Value
**Subjects (N)**	51	185	-	45	65	-
**Sex (Male) (%)**	16 (66.7%) (n = 24) *	124 (67.0%)	0.985	28 (80.0%)	50 (78.0%)	0.797
**Age (years)**	2.58 (3.06) (n = 24) *	8.19 (4.67)	**0.001**	28.9 (8.87)	31.5 (9.11)	0.182
**Blood groups (cases) and ALL immunophenotypes**						
**Blood groups**	**Pediatric**			**Adults**	***p* value**	
**A**	21.0%			26.4%	0.688	
**B**	47.2%			34.0%	0.399	
**AB**	4.4%			11.3%	0.391	
**O**	27.5%			28.3%	1.000	
**B-cell ALL**	142 (77%)			47 (72.3%)	0.874	
**T-cell ALL**	43 (23%)			18 (27.7%)	0.694	
**Cytogenetic data of cases**						
**Group**	**Immunophenotype**			**Cytogenetic abnormality**		
**Pediatric**	B-cell ALL			Hyperdiploidy, Hyperdiploidy with other abnormalities (add X, 4,6,7) del 6q21q27		
	T-cell ALL			Mosaicism Hypodiploidy with add 1p36.6 Hypodiploidy with t(9;15)(p13;q11.2)		
**Adults**	B-cell ALL			Hyperdiploidy with other abnormality del 9p22p24, del 9p13p24, del 9q24.3 add 9p23, 10q24.3 t(1;19)(q25;p13.3),t(9;22)(q34;q11.2),4q12 gene translocation, FIP1L1-PDGFRA fusion. dup 1q25q44		
	T-cell ALL			del 9p21p24		

Values are either N (%) or Mean (SE). *p* values are calculated from chi square or Mann–Whitney U test, * numbers in brackets represent known data.

**Table 3:** Logistic regression models showing association of telomere modulating genes (CTC1, OBFC1, TERT) with ALL.

	Pediatric						Adults					
	Association of Genes (CTC1, OBFC1, TERT) with ALL											
	A						B					
	Model 1 (Univariate Analysis)			Model 2 (Multivariate Analysis)			Model 1 Univariate Analysis			Model 2 Multivariate Analysis		
**Predictors**	**β-Estimate**	**SE**	***p* Value**	**β-Estimate**	**SE**	***p* Value**	**β-Estimate**	**SE**	***p* Value**	**β-Estimate**	**SE**	***p* Value**
CTC1	−0.045	0.009	**3.40 × 10^−6^**	−0.013	0.007	**0.040**	0.071	0.022	**0.002**	0.053	0.023	**0.025**
OBFC1	−0.017	0.011	0.127	−0.003	0.008	0.696	0.028	0.018	0.129	0.032	0.021	0.133
TERT	−0.010	0.010	0.335	−0.001	0.006	0.900	0.018	0.011	0.115	0.013	0.013	0.314
	**Combined association of genes with ALL**											
	**C**						**D**					
	**Model 3** **Multivariate Analysis**			**Model 4** **Multivariate Analysis**			**Model 3** **Multivariate Analysis**			**Model 4** **Multivariate Analysis**		
CTC1	−0.042	0.016	**0.010**	−0.040	0.012	**0.002**	0.097	0.033	**0.005**	0.105	0.037	**0.009**
OBFC1	0.002	0.020	0.907	0.027	0.014	0.056	−0.036	0.029	0.225	0.006	0.038	0.878
TERT	0.001	0.014	0.982	0.003	0.009	0.686	0.037	0.029	0.039	0.011	0.025	0.671

SE: Standard error. All the multivariable analysis except Model 3 were adjusted for age and sex. Statistically significant data are shown in bold.

**Table 4:** Logistic regression models showing association of telomere length with ALL.

	Pediatric			Adults		
	Association of TEL with ALL					
	A			D		
	Model 5 Univariate Analysis			Model 5 Univariate Analysis		
**Predictors**	**β-Estimate**	**SE**	***p* Value**	**β-Estimate**	**SE**	***p* Value**
TEL	0.112	0.108	0.305	0.576	0.511	0.267
	**Combined Effect of Genes and TEL with ALL**					
	**B**			**E**		
	**(Model 6)** Multivariate Analysis *			**(Model 6)** Multivariate Analysis *		
TEL	0.471	0.541	0.394	−1.044	2.319	0.683
CTC1	−0.026	0.037	0.480	0.193	0.212	0.429
OBFC1	−0.109	0.053	0.053	−0.029	0.187	0.888
TERT	0.059	0.039	0.149	0.022	0.850	0.813
	**C**			**F**		
	**(Model 7)** Multivariate Analysis **			**(Model 7)** Multivariate Analysis **		
TEL	0.947	0.521	0.103	−3.157	0.592	0.118
CTC1	0.096	0.075	0.232	0.139	0.049	0.218
OBFC1	−0.011	0.058	0.085	0.081	0.044	0.321
TERT	0.018	0.035	0.627	0.001	0.019	0.975

SE: Standard error, * = unadjusted for age and sex, ** = adjusted for age and sex. Statistically significant *p* value is shown in bold.

**Table 5:** Logistic regression models showing association of genes with ALL subtypes.

	Pediatrics						Adults					
A. Association of Genes with B-cell ALL												
Predictors	Univariate Analysis			Multivariate Analysis *			Univariate Analysis			Multivariate Analysis *		
	**β-Estimate**	**SE**	***p* Value**	**β-Estimate**	**SE**	***p* Value**	**β-Estimate**	**SE**	***p* Value**	**β-Estimate**	**SE**	***p* Value**
CTC1	−0.057	0.017	**0.002**	−0.028	0.019	0.148	0.056	0.043	0.208	0.118	0.059	0.059
OBFC1	−0.008	0.018	0.664	0.007	0.021	0.734	−0.039	0.044	0.366	−0.009	0.067	0.886
TERT	0.022	0.016	0.176	0.001	0.017	0.964	−0.093	0.043	**0.036**	−0.092	0.044	**0.043**
**B. Association of Genes with T-cell ALL**												
CTC1	−0.050	0.018	**0.008**	−0.039	0.036	0.301	−0.068	0.063	0.936	0.057	0.068	0.408
OBFC1	−0.017	0.017	0.321	0.032	0.035	0.372	−0.014	0.039	0.721	0.003	0.068	0.996
TERT	−0.019	0.016	0.233	−0.047	0.022	0.054	−0.085	0.042	0.053	−0.093	0.047	0.057
**C. Combined Effect of Genes with B-cell ALL**												
	**Pediatrics**						**Adults**					
	**Multivariate Analysis**			Multivariate Analysis *			**Multivariate Analysis**			Multivariate Analysis *		
CTC1	−0.057	0.023	**0.016**	−0.080	0.028	**0.008**	0.060	0.065	0.375	0.119	0.157	0.481
OBFC1	−0.032	0.030	0.281	0.024	0.030	0.426	−0.056	0.048	0.269	−0.059	0.010	0.578
* TERT	0.047	0.023	**0.040**	0.027	0.021	0.205	−0.095	0.067	0.188	−0.050	0.130	0.720
**D. Combined Effect of Genes with T-cell ALL**												
CTC1	−0.039	0.022	0.082	−0.057	0.026	**0.040**	−0.024	0.064	0.721	0.100	0.182	0.609
OBFC1	−0.020	0.030	0.499	0.087	0.028	**0.010**	0.004	0.048	0.938	−0.078	0.077	0.371
TERT	−0.013	0.026	0.627	−0.070	0.024	**0.012**	−0.086	0.060	0.179	−0.134	0.116	0.313

Results are adjusted with age and sex, SE= standard error, all significant results are represented in bold font. * Adjusted for age and sex.

## Data Availability

Data presented in this study are available on fair request to the corresponding author.

## Supplementary Materials

The following supporting information can be downloaded at: https://www.mdpi.com/article/10.3390/jcm11195780/s1.

## References

[1] Jebaraj B.M.C., Stilgenbauer S. (2021). Telomere Dysfunction in Chronic Lymphocytic Leukemia. Front. Oncol..

[2] Allegra A., Innao V., Penna G., Gerace D., Allegra A.G., Musolino C. (2017). Telomerase and telomere biology in hematological diseases: A new therapeutic target. Leuk. Res..

[3] de Lange T. (2018). Shelterin-mediated telomere protection. Annu. Rev. Genet..

[4] Tardat M., Déjardin J. (2018). Telomere chromatin establishment and its maintenance during mammalian development. Chromosoma.

[5] Jafri M.A., Ansari S.A., Alqahtani M.H., Shay J.W. (2016). Roles of telomeres and telomerase in cancer, and advances in telomerase-targeted therapies. Genome Med..

[6] Ghimire S., Hill C.V., Sy F.S., Rodriguez R. (2019). Decline in telomere length by age and effect modification by gender, allostatic load and comorbidities in National Health and Nutrition Examination Survey (1999–2002). PLoS ONE.

[7] Lin J., Cheon J., Brown R., Coccia M., Puterman E., Aschbacher K., Blackburn E.H. (2016). Systematic and cell type-specific telomere length changes in subsets of lymphocytes. J. Immunol. Res..

[8] Ackermann S., Fischer M. (2019). Telomere Maintenance in Pediatric Cancer. Int. J. Mol. Sci..

[9] Trybek T., Kowalik A., Góźdź S., Kowalska A. (2020). Telomeres and telomerase in oncogenesis (Review). Oncol. Lett..

[10] Nogueira B.M.D., Machado C.B., Montenegro R.C., De Moraes M.E.A., Moreira-Nunes C.A. (2020). Telomere Length and Hematological Disorders: A Review. In Vivo.

[11] Li Y., Tergaonkar V. (2016). Telomerase reactivation in cancers: Mechanisms that govern transcriptional activation of the wild-type vs. mutant *TERT* promoters. Transcription.

[12] Tomita K. (2018). How long does telomerase extend telomeres? Regulation of telomerase release and telomere length homeostasis. Curr. Genet..

[13] Maciejowski J., De Lange T. (2017). Telomeres in cancer: Tumour suppression and genome instability. Nat. Rev. Mol. Cell Biol..

[14] Shastrula P.K., Rice C.T., Wang Z., Lieberman P.M., Skordalakes E. (2018). Structural and functional analysis of an OB-fold in human Ctc1 implicated in telomere maintenance and bone marrow syndromes. Nucleic Acids Res..

[15] Barazas M., Annunziato S., Pettitt S.J., de Krijger I., Ghezraoui H., Roobol S.J., Lutz C., Frankum J., Song F.F., Brough R. (2018). The CST Complex Mediates End Protection at Double-Strand Breaks and Promotes PARP Inhibitor Sensitivity in BRCA1-Deficient Cells. Cell Rep..

[16] Huang C., Dai X., Chai W. (2012). Human Stn1 protects telomere integrity by promoting efficient lagging-strand synthesis at telomeres and mediating C-strand fill-in. Cell Res..

[17] Rice C., Skordalakes E. (2016). Structure and function of the telomeric CST complex. Comput. Struct. Biotechnol. J..

[18] Stewart J.A., Wang Y., Ackerson S.M., Schuck P.L. (2018). Emerging roles of CST in maintaining genome stability and human disease. Front. Biosci..

[19] Hom R.A., Wuttke D.S. (2017). Human CST Prefers G-Rich but Not Necessarily Telomeric Sequences. Biochemistry.

[20] Fan H.-C., Chang F.-W., Tsai J.-D., Lin K.-M., Chen C.-M., Lin S.-Z., Liu C.-A., Harn H.-J. (2021). Telomeres and Cancer. Life.

[21] Luo Y.M., Xia N.X., Yang L., Li Z., Yang H., Yu H.J., Liu Y., Lei H., Zhou F.X., Xie C.H. (2014). CTC1 increases the radioresistance of human melanoma cells by inhibiting telomere shortening and apoptosis. Int. J. Mol. Med..

[22] Han P., Dang Z., Shen Z., Dai H., Bai Y., Li B., Shao Y. (2019). Association of SNPs in the OBFC1 gene and laryngeal carcinoma in Chinese Han male population. Int. J. Clin. Oncol..

[23] Calado R.T., Young N.S. (2009). Telomere diseases. N. Engl. J. Med..

[24] Reddel R.R. (2014). Telomere Maintenance Mechanisms in Cancer: Clinical Implications. Curr. Pharm. Des..

[25] Wang L., Ma T., Liu W., Li H., Luo Z., Feng X. (2022). Pan-Cancer Analyses Identify the CTC1-STN1-TEN1 Complex as a Protective Factor and Predictive Biomarker for Immune Checkpoint Blockade in Cancer. Front Genet..

[26] Desmeules P., Dufour M., Fernandes M.J. (2009). A rapid flow cytometry assay for the assessment of calcium mobilization in human neutrophils in a small volume of lysed whole-blood. J. Immunol. Methods.

[27] Cawthon R.M. (2002). Telomere measurement by quantitative PCR. Nucleic Acids Res..

[28] R Core Team (2020). R: A Language and Environment for Statistical Computing.

[29] Paul S., Kantarjian H., Jabbour E.J. (2016). Adult Acute Lymphoblastic Leukemia. Mayo Clin. Proc..

[30] Maheswaran R., Morley N. (2018). Incidence, socioeconomic deprivation, volume-outcome and survival in adult patients with acute lymphoblastic leukaemia in England. BMC Cancer.

[31] Tavasolian F., Abdollahi E., Vakili M., Amini A. (2014). Relationship between ABO blood group and Acute Lymphoblastic Leukemia. Iran. J. Pediatr. Hematol. Oncol..

[32] Ghali H.H., Nayeef A.M., Hameed A.H., Fawzi G.M. (2017). Relationship between ABO and Rh Blood Groups with Childhood Acute Lymphoblastic Leukemia. IOSR J. Res. Method Educ. IOSRJRME.

[33] Mushtaq N., Fadoo Z., Naqvi A. (2013). Childhood acute lymphoblastic leukaemia: Experience from a single tertiary care facility of Pakistan. J. Pak. Med. Assoc..

[34] Terwilliger T., Abdul-Hay M. (2017). Acute lymphoblastic leukemia: A comprehensive review and 2017 update. Blood Cancer J..

[35] Safaei A., Shahryari J., Farzaneh M.R., Tabibi N., Hosseini M. (2013). Cytogenetic Findings of Patients with Acute Lymphoblastic Leukemia in Fars Province. Iran. J. Med. Sci..

[36] Alonso C.N., Rossi J.G., Bernasconi A.R., Rampazzi M.A., Felice M.S., Rubio P.L., Eberle S.E., Medina A., Gallego M.S., Coccé M.C. (2015). Cytogenetic and Molecular Findings in Children with Acute Lymphoblastic Leukemia: Experience of a Single Institution in Argentina. Mol. Syndr..

[37] Reddy P., Shankar R., Koshy T., Radhakrishnan V., Ganesan P., Jayachandran P.K., Dhanushkodi M., Mehra N., Krupashankar S., Manasa P. (2019). Evaluation of Cytogenetic Abnormalities in Patients with Acute Lymphoblastic Leukemia. Indian J. Hematol. Blood Transfus..

[38] Carroll A.J., Shago M., Mikhail F.M., Raimondi S.C., Hirsch B.A., Loh M.L., Raetz E.A., Borowitz M.J., Wood B.L., Maloney K.W. (2019). Masked hypodiploidy: Hypodiploid acute lymphoblastic leukemia (ALL) mimicking hyperdiploid ALL in children: A report from the Children’s Oncology Group. Cancer Genet..

[39] Chaturvedi A., Shetty D., Ghogale S.G., Deshpande N., Badrinath Y., Chatterjee G., Girase K., Sriram H., Khanka T., Mishra C. (2022). Detecting hypodiploidy with endoreduplication and masked hypodiploidy in B-cell acute lymphoblastic leukemia using multicolor flow cytometry. Cytom. Part B Clin. Cytom..

[40] Okamoto K., Seimiya H. (2019). Revisiting Telomere Shortening in Cancer. Cells.

[41] Vyas C.M., Ogata S., Reynolds C.F., Mischoulon D., Chang G., Cook N.R., Manson J.E., Crous-Bou M., De Vivo I., Okereke O.I. (2021). Telomere length and its relationships with lifestyle and behavioural factors: Variations by sex and race/ethnicity. Age Ageing.

[42] Walsh K.M., Whitehead T.P., de Smith A.J., Smirnov I.V., Park M., Endicott A.A., Francis S.S., Codd V., Samani N.J., Metayer C. (2016). Common genetic variants associated with telomere length confer risk for neuroblastoma and other childhood cancers. Carcinogenesis.

[43] Wang Y., Fang M., Sun X., Sun J. (2010). Telomerase activity and telomere length in acute leukemia: Correlations with disease progression, subtypes and overall survival. Int. J. Lab. Hematol..

[44] Eskandari E., Hashemi M., Naderi M., Bahari G., Safdari V., Taheri M. (2018). Leukocyte Telomere Length Shortening, hTERT Genetic Polymorphisms and Risk of Childhood Acute Lymphoblastic Leukemia. Asian Pac. J. Cancer Prev..

[45] Lansdorp P. (2017). Maintenance of telomere length in AML. Blood Adv..

[46] Feng X., Hsu S.-J., Kasbek C., Chaiken M., Price C.M. (2017). CTC1-mediated C-strand fill-in is an essential step in telomere length maintenance. Nucleic Acids Res..

[47] Zaug A.J., Lim C.J., Olson C.L., Carilli M.T., Goodrich K.J., Wuttke D.S., Cech T.R. (2021). CST does not evict elongating telomerase but prevents initiation by ssDNA binding. Nucleic Acids Res..

[48] Savage S.A., Bertuch A.A. (2010). The genetics and clinical manifestations of telomere biology disorders. Genet. Med..

[49] Karow A., Haubitz M., Leibundgut E.O., Helsen I., Preising N., Steiner D., Dantonello T., Ammann R., Roessler J., Kartal-Kaess M. (2021). Targeting Telomere Biology in Acute Lymphoblastic Leukemia. Int. J. Mol. Sci..

[50] Tabori U., Dome J.S. (2007). Telomere Biology of Pediatric Cancer. Cancer Investig..

[51] Angstadt A.Y., Thayanithy V., Subramanian S., Modiano J.F., Breen M. (2012). A genome-wide approach to comparative oncology: High-resolution oligonucleotide aCGH of canine and human osteosarcoma pinpoints shared microaberrations. Cancer Genet..

[52] Giaccherini M., Macauda A., Sgherza N., Sainz J., Gemignani F., Maldonado J.M.S., Jurado M., Tavano F., Mazur G., Jerez A. (2020). Genetic polymorphisms associated with telomere length and risk of developing myeloproliferative neoplasms. Blood Cancer J..

[53] Ojha J., Codd V., Nelson C.P., Samani N.J., Smirnov I.V., Madsen N.R., Hansen H.M., de Smith A.J., Bracci P.M., Wiencke J.K. (2016). Genetic Variation Associated with Longer Telomere Length Increases Risk of Chronic Lymphocytic Leukemia. Cancer Epidemiol. Biomarkers Prev..

[54] Cardinale A., Cantalupo S., Lasorsa V.A., Montella A., Cimmino F., Succoio M., Vermeulen M., Baltissen M.P., Esposito M., Avitabile M. (2022). Functional annotation and investigation of the 10q24.33 melanoma risk locus identifies a common variant that influences transcriptional regulation of *OBFC1*. Hum. Mol. Genet..

[55] Wang F., Stewart J., Price C.M. (2014). Human CST abundance determines recovery from diverse forms of DNA damage and replication stress. Cell Cycle.

[56] Kibriya M.G., Raza M., Kamal M., Haq Z., Paul R., Mareczko A., Pierce B.L., Ahsan H., Jasmine F. (2022). Relative Telomere Length Change in Colorectal Carcinoma and Its Association with Tumor Characteristics, Gene Expression and Microsatellite Instability. Cancers.

[57] Çoğulu O., Kosova B., Karaca E., Gunduz C., Ozkinay F., Aksoylar S., Gülen H., Kantar M., Oniz H., Karapinar D. (2004). Evaluation of Telomerase mRNA (hTERT) in Childhood Acute Leukemia. Leuk. Lymphoma.

[58] Rudant J., Orsi L., Bonaventure A., Goujon-Bellec S., Baruchel A., Petit A., Bertrand Y., Nelken B., Pasquet M., Michel G. (2015). ARID5B, IKZF1 and Non-Genetic Factors in the Etiology of Childhood Acute Lymphoblastic Leukemia: The ESCALE Study. PLoS ONE.

[59] Pereira F.A.C., Mirra A.P., Latorre M.D.R.D.D.O., De Assunção J.V. (2017). Fatores de risco ambientais e leucemia linfobla?stica aguda na infa?ncia. Rev. Cienc. Salud.

[60] Poncet D., Belleville A., de Roodenbeke C.T., de Climens A.R., Ben Simon E., Merle-Beral H., Callet-Bauchu E., Salles G., Sabatier L., Delic J. (2008). Changes in the expression of telomere maintenance genes suggest global telomere dysfunction in B-chronic lymphocytic leukemia. Blood.

[61] Sallan S.E. (2006). Myths and lessons from the adult/pediatric interface in acute lymphoblastic leukemia. Hematol. Am. Soc. Hematol. Educ. Program.

[62] Chaudhury S., O’Connor C., Cañete A., Bittencourt-Silvestre J., Sarrou E., Prendergast A., Choi J., Johnston P., Wells C.A., Gibson B. (2018). Age-specific biological and molecular profiling distinguishes peadiatric from adult acute myeloid leukemias. Nat. Commun..

