# Supplementary material for: Transcriptomic Analysis of Conserved Telomere Maintenance Component 1 (CTC1) and Its Association with Leukemia
Source: J Clin Med. 2022 Sep 29;11(19):5780. doi: 10.3390/jcm11195780 (PMC9571731; doi:10.3390/jcm11195780)

Supplementary Materials

Material and Methods

Statistical Analysis

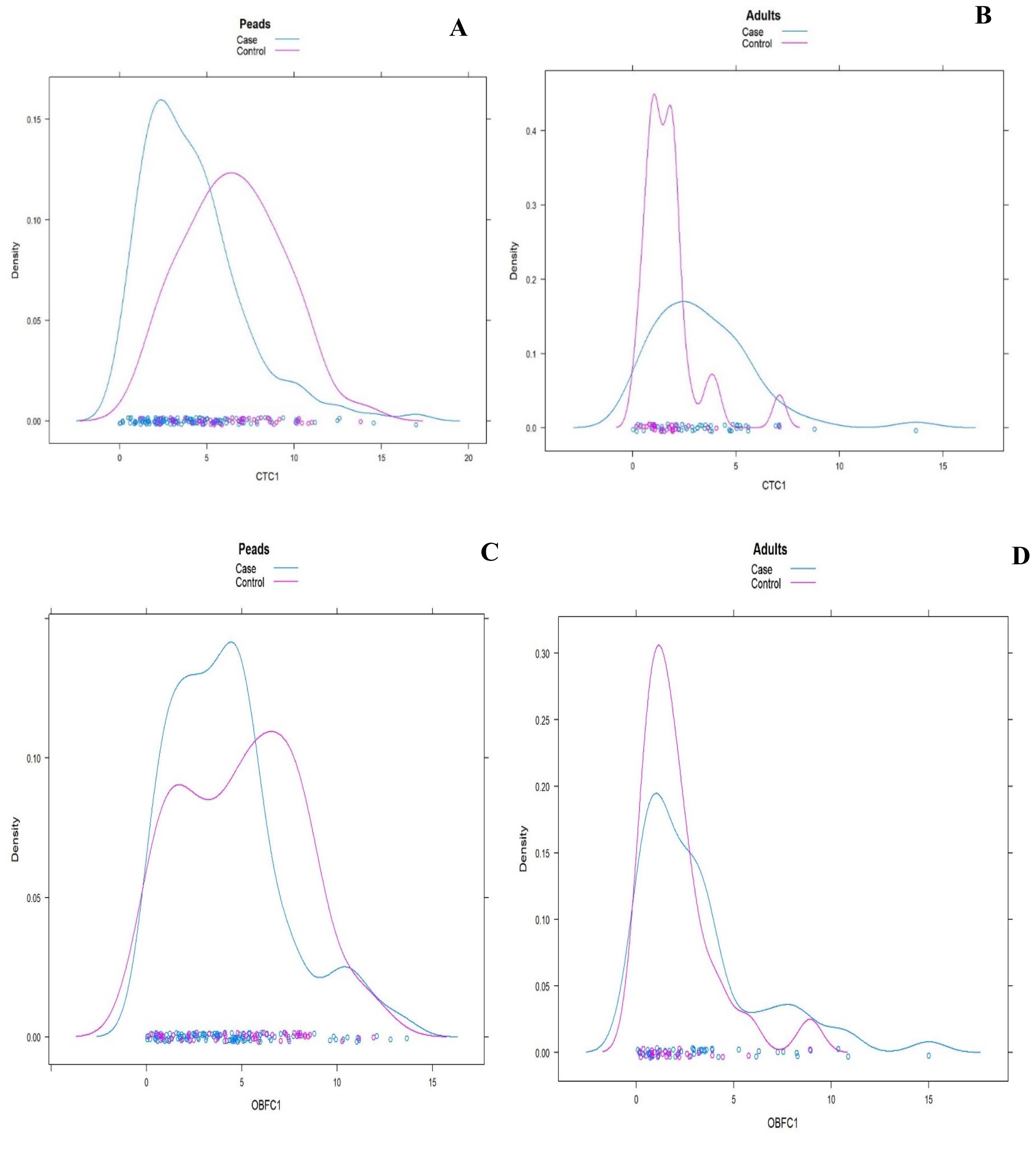

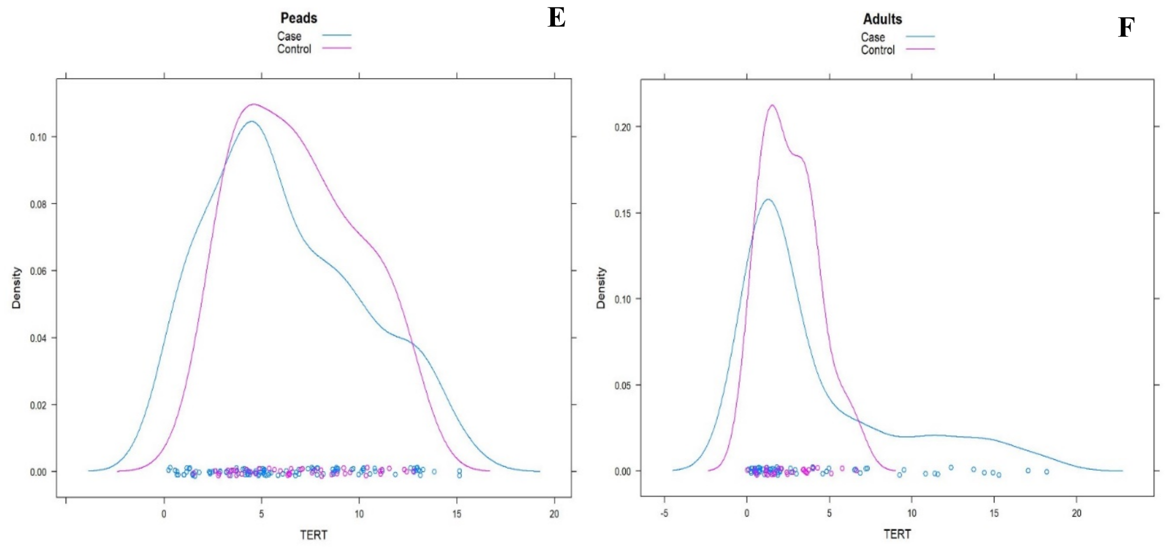

Supplementary Figure S1. (**A–F**): Density plots showing distribution of ΔCt values of CTC1, OBFC1 and TERT genes in control and cases of pediatric and adult groups. A, C, E, = Pediatric groups and B, D, F = Adult groups.

Results:

Correlation between the genes (CTC1, OBFC1, TERT) expression and Telomere length in pediatric and adult groups:

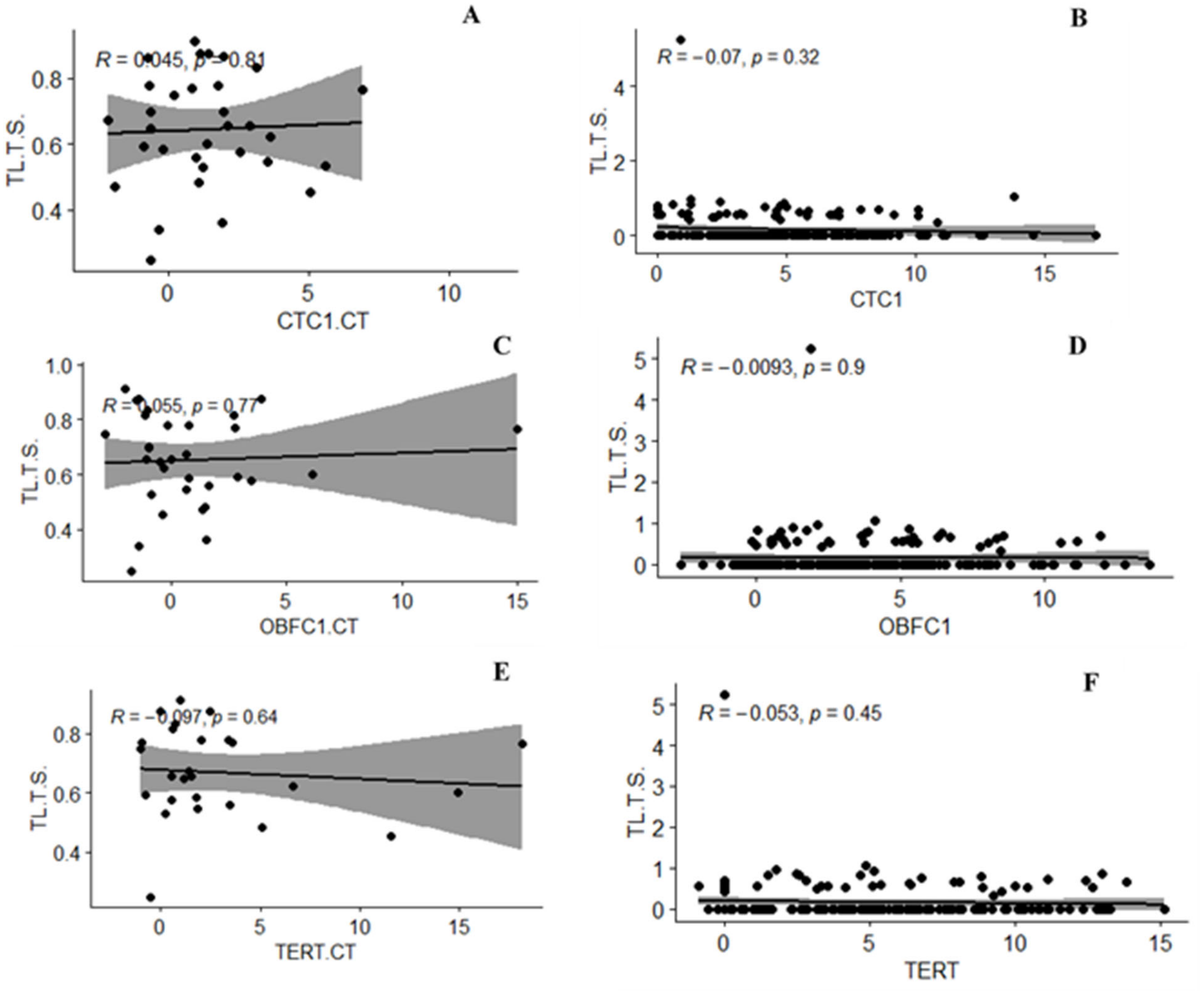

Supplementary Figure S2. (**A–F**): Correlation between genes and telomere length (A-B) CTC1 and TEL, (C-D) OBFC1 and TEL, (E-F) TERT and TEL. A, C, E = Adult groups and B, D, F = Pediatric groups. Non-significant (p value > 0.05) correlation has been noted between the genes and telomere length in pediatric and adult groups.

Flow Chart showing statistical models in Table 3

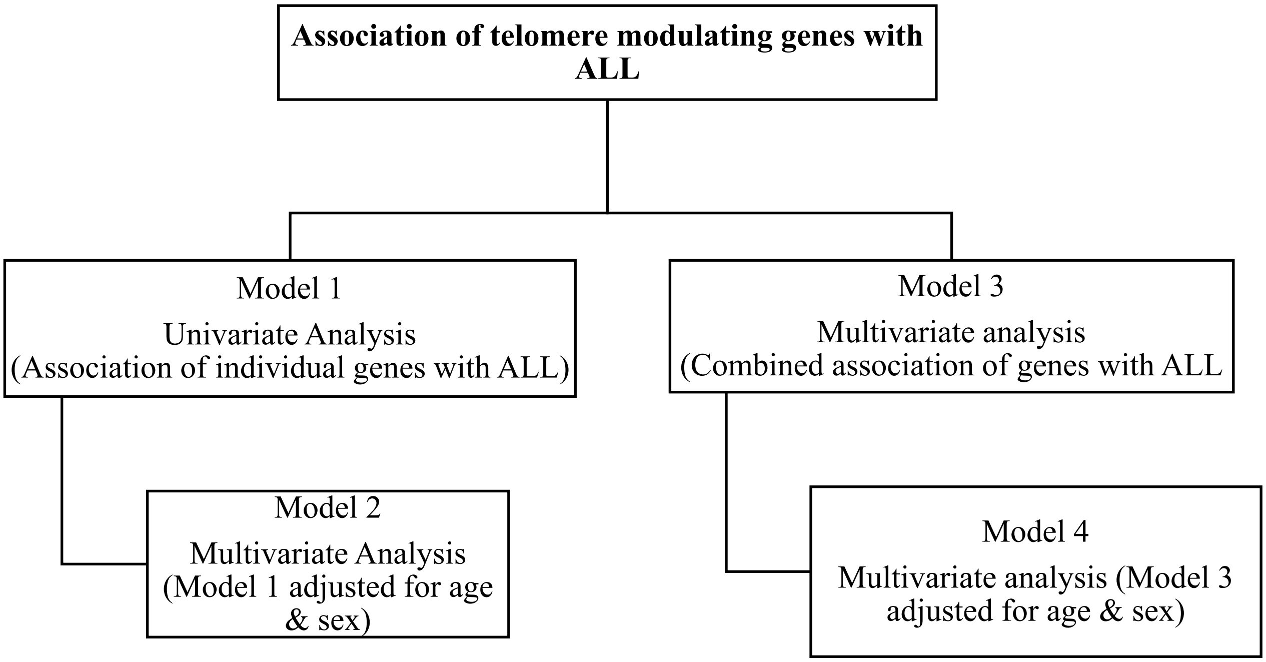

Flow chart showing statistical models in Table 4

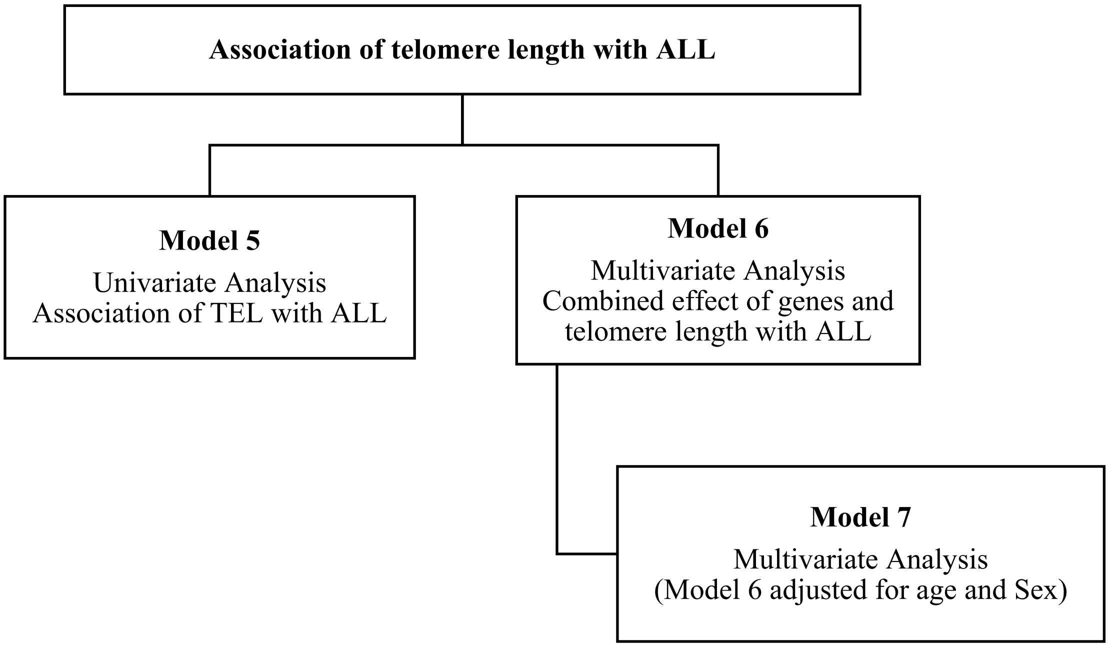

Supplement: Supplementary file 1 [file jcm-11-05780-s001.zip › jcm-1910838-supplementary.pdf]
